# GATA3 Truncation Mutants Alter EMT Related Gene Expression *via* Partial Motif Recognition in Luminal Breast Cancer Cells

**DOI:** 10.3389/fgene.2022.820532

**Published:** 2022-01-28

**Authors:** Mika Saotome, Deepak Balakrishnan Poduval, Renju Nair, Mikhala Cooper, Motoki Takaku

**Affiliations:** Department of Biomedical Sciences, University of North Dakota School of Medicine and Health Sciences, Grand Forks, ND, United States

**Keywords:** breast cancer, GATA3, GATA-3, somatic mutations, luminal breast cancer, epithelial-to-mesenchymal transition

## Abstract

GATA3 is known to be one of the most frequently mutated genes in breast cancer. More than 10% of breast tumors carry mutations in this gene. However, the functional consequence of GATA3 mutations is still largely unknown. Clinical data suggest that different types of GATA3 mutations may have distinct roles in breast cancer characterization. In this study, we have established three luminal breast cancer cell lines that stably express different truncation mutants (X308 splice site deletion, C321 frameshift, and A333 frameshift mutants) found in breast cancer patients. Transcriptome analysis identified common and distinct gene expression patterns in these GATA3 mutant cell lines. In particular, the impacts on epithelial-to-mesenchymal transition (EMT) related genes are similar across these mutant cell lines. Chromatin localization of the mutants is highly overlapped and exhibits non-canonical motif enrichment. Interestingly, the A333 frameshift mutant expressed cells displayed the most significant impact on the GATA3 binding compared to X308 splice site deletion and C321fs mutants expressed cells. Our results suggest the common and different roles of GATA3 truncation mutations during luminal breast cancer development.

## Introduction

Breast cancer is the most common cancer among women in the U.S. and the second leading cause of cancer-related deaths. It was projected that there would be about 284,200 new cases of invasive breast cancer and the deaths of approximately 43,600 women in the U.S. from breast cancer in 2021 (American Cancer Society, 2018). GATA3 is a reliable biomarker for breast carcinomas and is frequently used to determine the tissue of origin to confirm a diagnosis (Jensen et al., 2002; Garcia-Closas et al., 2007; Bertucci et al., 1999; Liu et al., 2016; Hoch et al., 1999; Mehra et al., 2005; Sørlie et al., 2003; Chou et al., 2010; Takaku et al., 2015). Recent large-scale molecular profiling of breast carcinomas identified frequent mutations in GATA3 (Usary et al., 2004; The Cancer Genome Atlas Network, 2012). GATA3 is a transcription factor and is known to be involved in multiple developmental pathways and human diseases such as normal mammary gland development, breast cancer progression, and T cell differentiation (Zheng and Flavell, 1997; Kouros-Mehr et al., 2006; Chou et al., 2010). In breast cancer, it has been shown that the cooperative action between GATA3 and its cofactors Estrogen Receptor alpha (ER-*α*) and FOXA1 is important for the luminal breast cancer characterization (Kong et al., 2011; Theodorou et al., 2013; Takaku et al., 2020). Based on the METABRIC cohort (Pereira et al., 2016), among 1980 patient cases, 230 breast carcinomas harbored GATA3 mutations (∼11.6%). They observed 75% of the mutations in luminal tumors (47% in luminal A, 28% in luminal B). These data suggest that approximately 50,000 new cases of invasive breast cancer in the U.S. will carry GATA3 mutations. While patient genomic data suggests GATA3 mutations as cancer drivers, the functional consequences of GATA3 mutations in breast cancer are underexplored (Adomas et al., 2014; Mair et al., 2016; Gustin et al., 2017; Emmanuel et al., 2018; Takaku et al., 2018). More importantly, the frequency of somatic mutations in GATA3 was even higher in the metastatic breast cancer cohort (Bertucci et al., 2019). GATA3 mutant breast cancer patients had lung, lymph nodes, and brain metastases (Bruna et al., 2016; Bertucci et al., 2019). The GATA3 mutant tumors tend to occur in younger patients (<45 years of age) (Azim et al., 2015; Griffith et al., 2018). The development of tamoxifen resistance correlates with the GATA3 gene silencing (Feng et al., 2014; Bi et al., 2020). These facts highlight the importance of the functional study of GATA3 and its mutations in breast cancer.

We previously identified that patients carrying GATA3 mutations have diverse clinical features. More than 70% of cases are small nucleotide deletions or insertions (indel), while less than 30% are missense mutations. By classifying the GATA3 indel mutations into four groups, we observed distinct clinical features (Takaku et al., 2018). Somatic mutations found in the GATA3 second zinc-finger domain (ZnFn2) are associated with poorer patient outcomes and low survival rates. The ZnFn2 domain is known to be important for the DNA binding and transcription activation activities of the GATA3 protein. ZnFn2 mutations are predominantly found in luminal B breast tumors, while splice site mutations are frequently found in luminal A breast tumors and are associated with better patient survival. These distinct clinical features suggest the differential impacts of GATA3 mutations on breast cancer cells. However, the biological significance and consequences, including the cellular function of these mutants, are not well-studied (Usary et al., 2004; Gustin et al., 2017; Emmanuel et al., 2018; Takaku et al., 2018; Hruschka et al., 2020; Takaku et al., 2020). In this study, we have established three luminal breast cancer cell lines that stably express different GATA3 truncation mutants found in breast cancer. We explored the function of these mutants using genomic approaches. These results revealed the common and distinct impacts of GATA3 truncation mutants on luminal breast cancer cells.

## Materials and Methods

### Cell Line and Cell Culture

T47D cells (originally purchased from ATCC) were maintained in Gibco DMEM high glucose medium (Thermo) supplemented with 10% FBS (Atlanta). GATA3 mutant genes were generated by site-directed mutagenesis using the primers (Supplementary Figure S1A) and cloned into pHAGE lentiviral vectors (a kind gift from Dr. Guang Hu at NIEHS/NIH). The viruses were produced by transient transfection using 293T cells, psPAX2, and pMD2.G packaging vectors (psPAX2 and pMD2.G were gifts from Dr. Didier Trono, Addgene plasmid 12260, 12259). After infection, with lentiviruses encoding each GATA3 mutant, the stable cell pools were established by puromycin selection for 1 week. T47D cells infected with the lentivirus containing the pHAGE empty vector were used as a control cell line.

### Cell Proliferation Assay

T47D cells were resuspended in Gibco DMEM high glucose medium (Thermo) containing 10% FBS (Atlanta). Fifty thousand cells per well were plated in a 24-well plate. At Days 1, 3, and 5, cells were harvested and then counted by DeNovix CellDrop.

### RNA-Seq

RNA was purified by the Direct-zol RNA Miniprep Kit (Zymo). RNA-seq libraries were prepared by the UND genomics core using NEBNext Ultra II RNA-Seq Kit (NEB). Tapestation (Agilent technologies) was used to check the RNA integrity and library quality. Pooled libraries were sequenced in one lane Nova-Seq S4 as 150 bp paired-end reads. RNA-Seq data were analyzed using an in-house pipeline. In the first step, reads were trimmed based on quality, and adapters were removed using Trimmomatic (v0.39) (Bolger et al., 2014). Quality trimmed reads were aligned to the human genome (hg38), and raw read counts were obtained using featureCounts from Rsubread (Liao et al., 2014). Raw read counts were processed using a custom R script, and differential expression levels were quantified using DESeq2 (Love et al., 2014). Differentially expressed genes were annotated using clusterprofiler (Yu et al., 2012) and wikipathways (Slenter et al., 2018).

### ChIP-Seq and Peak Analysis

The details of the procedures were previously described (Takaku et al., 2018; Takaku et al., 2020). T47D cells were fixed with 1% formaldehyde at 37°C for 10 min with constant shaking. Fixed cells were incubated with hypotonic buffer containing 10 mM HEPES–NaOH pH 7.9, 10 mM KCl, 1.5 mM MgCl2, 340 mM sucrose, 10% glycerol, 0.5% Triton X-100 and Halt protease and phosphatase Inhibitor Cocktail (Thermo Fisher Scientific). After centrifugation, the cells were lysed with the sonication buffer containing 20 mM Tris-HCl pH 8.0, 2 mM EDTA, 0.5 mM EGTA, 0.5 mM PMSF, 5 mM sodium butyrate, 0.1% SDS and protease inhibitor cocktail. Nuclei were sonicated by Covaris S220 for 10 min.

Approximately 15 µg of chromatin was used for immunoprecipitation. For immunoprecipitation of the GATA3 mutants, 2.5 µg of anti-Ty1 antibody (Diagenode, C15200054) was used. For a total GATA3 pull-down, 2.5 µL of anti-GATA3 antibody (Cell Signaling, D13C9) was used in each assay. After overnight incubation, Protein G (for Ty1 antibody) or Protein A and G mix (for GATA3 antibody) Dynabeads (Thermo) were added to the chromatin solution. DNAs were purified by AMPure XP (Beckman Coulter). ChIP-seq libraries were prepared by NEXTFLEX Rapid DNA-Seq Kit.

ChIP-Seq reads were quality filtered and adapter trimmed using in-house scripts. The trimmed reads were mapped to the human genome (hg19) using Bowtie (version 1.2.2) (Langmead et al., 2009). Uniquely mapped reads were marked for duplicates with the Picard tools (Broad Institute, 2019). For subsequent analysis, paired reads were merged into single fragments, and coverage tracks were obtained using genomeCoverageBed from bedtools (v2.29.0) (Quinlan and Hall, 2010). ChIP-seq peaks were identified using HOMER (Heinz et al., 2010) with default parameters. Overlap between peak sets was determined using intersectBed from bedtools (v2.29.0). Differential binding events between samples were identified using EdgeR (Robinson et al., 2010) with FDR <0.05 and absolute fold change >1.5. Motif analysis was carried out using MEME-ChIP from The MEME Suite (Machanick and Bailey, 2011). The HOMER tool Perl script (findMotifs.pl) was used to find each motif frequency within the peaks. Read counts were collected in 20 bp bins, then normalized to 15 million total non-duplicate unique fragments per dataset for regions ±1 kb relative to peak midpoints.

## Results

### Establishment of GATA3 Truncation Mutant Cell Lines

**FIGURE 1:**
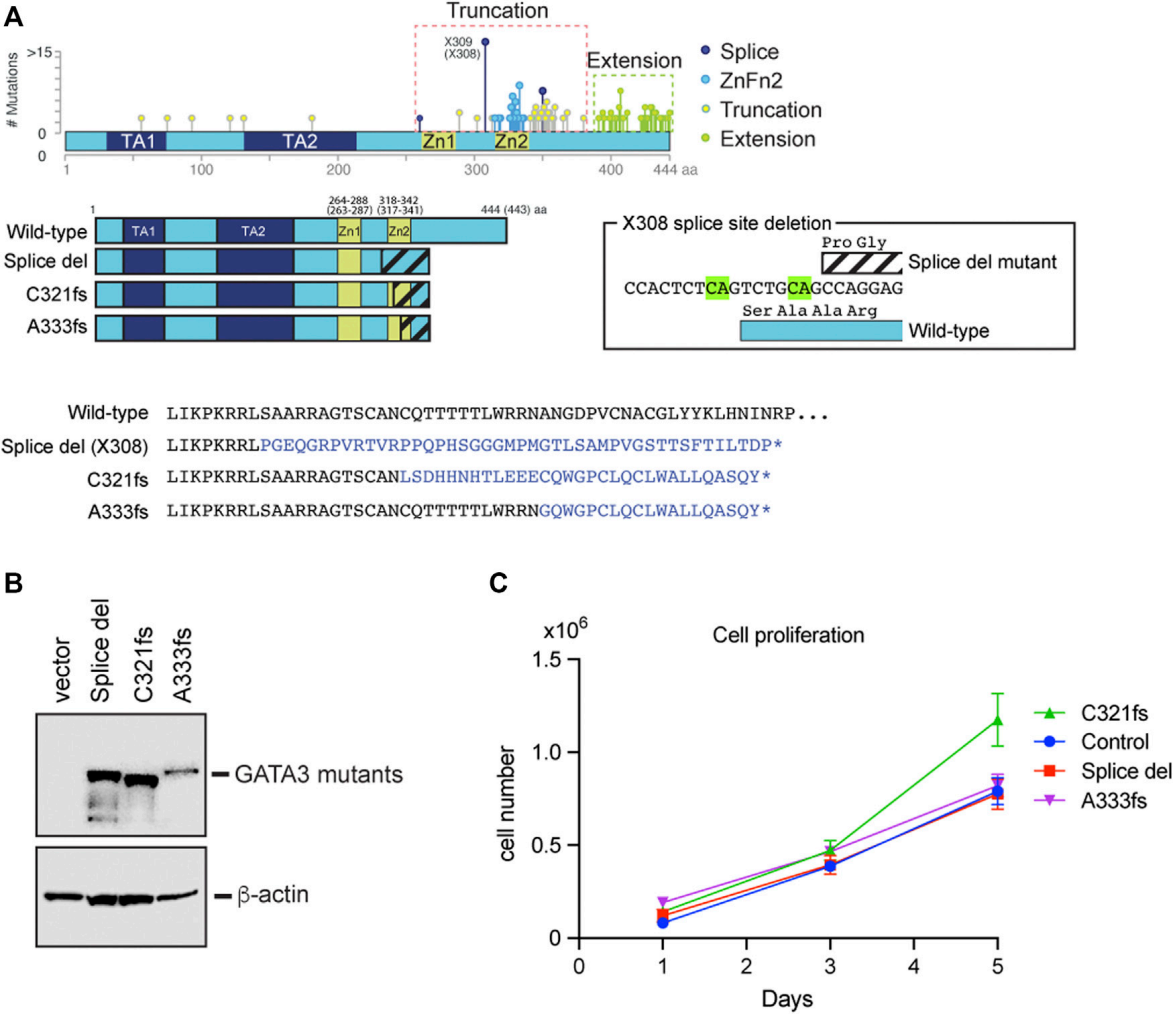
Establishment of GATA3 truncation mutant cell lines. **(A)** Distribution of GATA3 mutations found in METABRIC cohort. Based on the protein products and mutation sites, four groups were generated (Takaku et al., 2018). The secondary structure of GATA3 mutants used in this study is indicated. The altered amino acid residues are shown on the bottom. The X308 splice site deletion and its effect on the GATA3 protein are indicated in box. **(B)** Western blot showing GATA3 mutants. Anti-Ty1 antibody was used to detect each mutant. *β*-actin levels were used as input control. **(C)** Cell growth comparison between GATA3 mutant expressed T47D cells. The average cell number is indicated at each time point with each of their standard deviations (N = 3).

To better understand the roles of GATA3 mutants in breast cancer cells, we established three luminal breast cancer cell lines stably expressing X308 splice site deletion mutant (Splice del), C321 frameshift mutant (C321fs), and A333 frameshift mutant (A333fs), respectively (Figure 1A). Splice del mutation is one of the hot spot mutations found in breast cancer. Two nucleotide (CA) deletion at the splice acceptor site induces alternative splicing by using a new splice acceptor site seven nucleotides downstream. This results in a frameshift and protein truncation due to the immature stop codon (Takaku et al., 2015; Hruschka et al., 2020). The protein product entirely loses the original (wild-type) amino acid sequence of the ZnFn2 DNA binding domain. C321fs and A333fs mutations were found in the ZnFn2 domain and belong to the ZnFn2 mutation (Figure 1A). Again, these frameshift mutations result in protein truncation due to the immature stop, and they partially lack the ZnFn2 wild-type sequence. We exogenously expressed these mutants as 3xTy-1 fused protein in T47D cells by lentiviral transduction. After the positive clone selection by Puromycin, we looked at protein expression levels by western blot. The expression levels of Splice del and C321fs were similar, while the A333fs protein level was slightly lower (Figure 1B). All GATA3 mutant cell lines exhibited synonymous wild-type GATA3 expression levels compared to the control cell line (Supplementary Figure 1B). To investigate the roles of GATA3 mutants in cancer cell proliferation, we performed a cell proliferation assay. Although the cell number of the C321fs expressed cells on Day 5 was slightly higher, overall, the stable expression of these GATA3 truncation mutants did not exhibit a significant impact on T47D cell growth (Figure 1C).

### GATA3 Truncation Mutants Influence Luminal Breast Cancer Transcriptome

**FIGURE 2:**
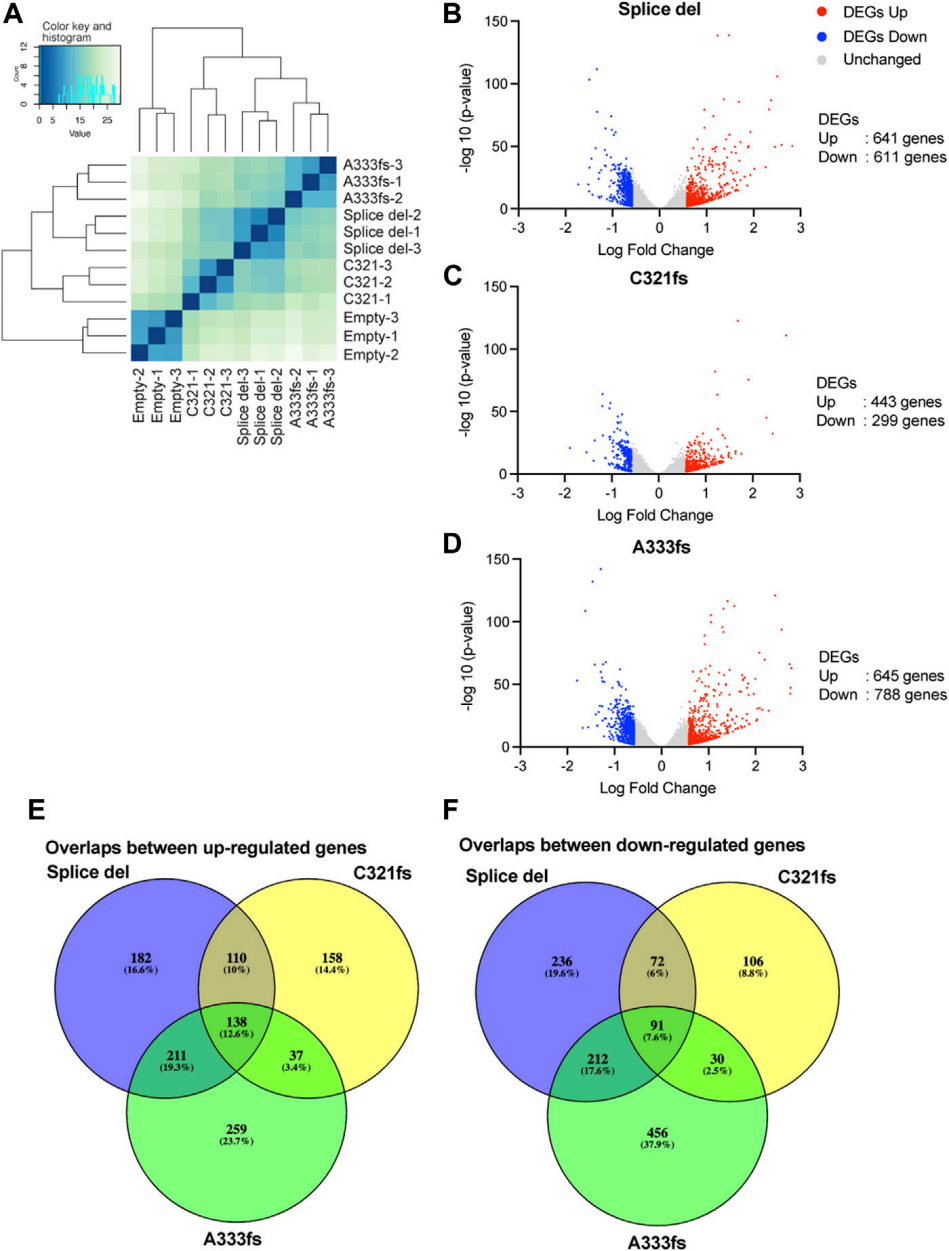
Gene expression analysis in GATA3 truncation mutant cells. **(A)** Clustered heatmap showing gene expression similarity between Splice del, C321fs, A333fs, and control T47D cells. The scale indicates Euclidean distance. **(B)** Volcano plot showing differential gene expression between Splice del mutant and control T47D cells. **(C)** Volcano plot showing differential gene expression in C321fs mutant cells. **(D)** Volcano plot showing differential gene expression in A333fs mutant expressed cells. FDR <0.05 and absolute fold change >1.5 were applied to define differentially expressed genes. Down- or up-regulated genes are highlighted in blue or red **(E,F)** Overlap analysis of up-**(E)** or down-**(F)** regulated genes between Splice del, C321fs, and A333fs mutant cells.

To identify the impacts of GATA3 mutant expression on gene expression, RNA-seq was performed for each of our established cell lines. The PCA plot and clustering analysis showed the high similarity between biological replicates (Figure 2A, Supplementary Figure S1C). The mutant expressing cell lines have distinct transcriptome profiles from the control T47D cells. Despite the weaker expression of A333fs mutant, A333fs and Splice del mutant T47D cells share similarities compared to C321fs mutant expressing cells or control cells. We then defined differentially expressed genes at a false discovery rate (FDR) < 0.01 and |fold change| > 1.5 (Supplementary Table S1). In Splice del mutant cells, there were 641 up-regulated genes and 611 down-regulated genes compared to control T47D cells (Figure 2B). C321fs mutant expressing cells presented 443 up- and 299 down-regulated genes (Figure 2C), while A333fs mutant expressing cells presented 645 up- and 788 down-regulated genes (Figure 2D). Among these differentially expressed genes, 138 were up-regulated across all three GATA3 mutant cell lines (Figure 2E), while 91 genes were commonly down-regulated in the mutant cells (Figure 2F). These data suggest that although Splice del, C321fs, and A333fs mutations result in a similar protein truncation, the impacts on luminal breast cancer transcriptome are partially different.

**FIGURE 3:**
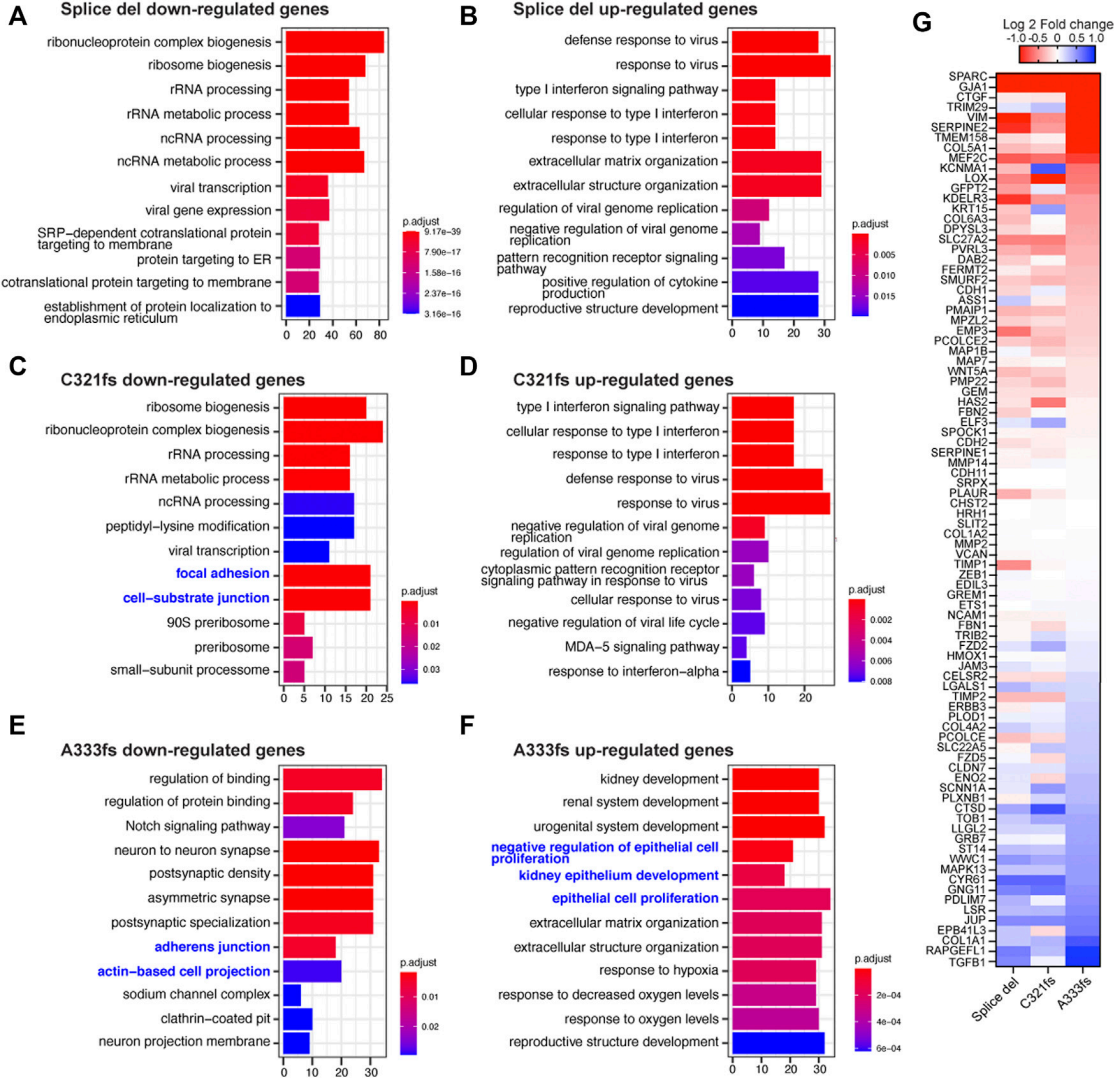
Biological pathways enriched in GATA3 mutant cells. **(A,B)** Top 12 significantly enriched pathways of down-**(A)** or up-**(B)** regulated genes in Splice del mutant T47D cells **(C,D)** Top 12 significantly enriched pathways of down-**(C)** or up-**(D)** regulated genes in C321fs mutant T47D cells **(E,F)** Top 12 significantly enriched pathways of down-**(E)** or up-**(F)** regulated genes in A333fs mutant T47D cells. Pathways related to EMT or MET are highlighted in blue. **(G)** Heatmap showing EMT-related gene expression in the mutant cells. Relative gene expression (Log two Fold change) against control T47D cells is shown. 91 universal EMT genes (Parsana et al., 2017) are used for the heatmap analysis.

To understand biological pathways enriched in the mutant expressed T47D cells, we performed GO term analysis. In C321fs and A333fs cells, the down-regulated genes were enriched for cell junction pathways (Figures 3C–E). Down-regulated genes in A333fs cells showed enrichment for the Notch signaling pathway and neuron-related pathways, while only Splice del and C321fs cells exhibited significant enrichment of ribosomal RNA processing pathways (Figures 3A,C,E). Among up-regulated genes, immune response pathways, including type I interferon and viral response networks, were enriched in Splice del and C321fs cells (Figures 3B,D). In A333fs cells, epithelial proliferation and development-related genes were enriched in up-regulated genes (Figure 3F). Since GATA3 is known to be a critical regulator of mammary epithelium development, EMT and its reverse process, mesenchymal-to-epithelial transition (MET), we specifically looked at genes involved in these pathways (Figure 3G). 91 universal EMT genes identified by Dr. Battle’s group (Lien et al., 2007) were used for this gene expression analysis. Although more distinct impacts on EMT-related genes were observed in A333fs cells, all three GATA3 mutant cells exhibit similar alteration patterns. For instance, some of the mesenchymal marker genes such as VIM, LOX, and SPARC (Lien et al., 2007; El-Haibi et al., 2012) were up-regulated in the mutant cell lines, while the expression levels of other EMT markers such as ZEB1 were not increased in the mutant cells, suggesting a partial EMT phenotype (Aiello and Kang, 2019). Taken together, these transcriptome analyses indicate that Splice del, C321fs, and A333fs mutants can modulate the expression levels of EMT-related genes.

### Non-Canonical Chromatin Binding by GATA3 Truncation Mutants

**FIGURE 4:**
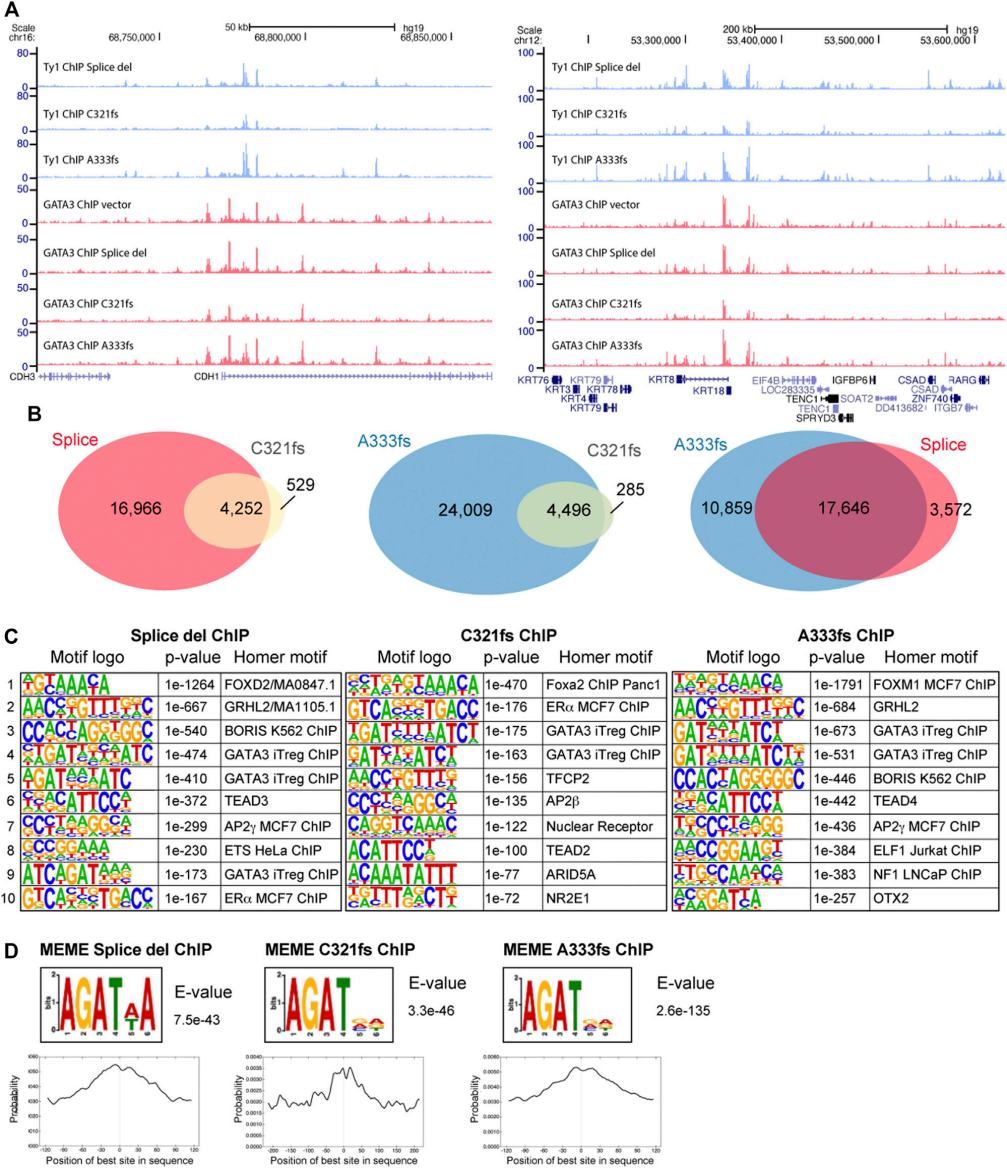
Chromatin distribution of GATA3 truncation mutants. **(A)** Genome browser tracks showing a representative genomic locus. The top three panels indicate Ty1 (GATA3 mutant) ChIP-seq. The bottom four panels indicate GATA3 ChIP-seq. GATA3 ChIP vector indicates the GATA3 ChIP-seq data from the control T47D cells (GATA3 wild-type). **(B)** Venn diagram showing peak overlap between Splice del, C321fs, and A333fs mutant ChIP-seq. **(C)** Top 10 HOMER *de novo* motifs enriched at each mutant peak. **(D)** GATA3 non-canonical binding motif identified by MEME-ChIP motif analysis. Motif distribution is shown on the bottom.

Using ChIP-seq, the chromatin distribution of GATA3 mutants was determined to identify how the truncation mutants alter transcription profiles in T47D cells. Since all mutants expressed as Ty1 epitope tag fusion proteins, we first performed Ty1 ChIP-seq specifically to map the chromatin localization of the mutants. Even though Splice del, C321fs, and A333fs mutations completely or partially lack the intact ZnFn_2_ domain, we observed many common peaks and that all three mutants bind to chromatin (Figure 4A). Peak call analysis by HOMER identified 21,218 Splice del peaks, 28,505 A333fs peaks, and 4,781 C321fs peaks. We further confirmed the mutant distributions and peak numbers by comparing the data between biological replicates (Supplementary Figures S2A–C). Since the fewer peaks in the C321fs ChIP-seq data suggest the weaker chromatin binding of C321fs mutant, we compared the signal enrichment in each mutant ChIP-seq data. Metaplot analysis of Ty1 ChIP-seq data clearly suggests that lower enrichment of C321fs compared to Splice del and A333fs (Supplementary Figures S2D,E). Peak overlap analysis indicated that the genomic distributions of Splice del, C321fs, and A333fs mutants were largely overlapped (Figures 4A,B). We also observed each mutant specific localization. Based on the peak overlap between A333fs and Splice del mutants, 10,859 peaks (∼37%) were uniquely enriched in A333fs ChIP-seq data, and 3,572 peaks (∼17%) did not overlapped with A333fs peaks. Since the ZnFn_2_ is known to be important for the consensus DNA motif binding, and we previously showed the altered DNA binding activities of R330fs and D336fs GATA3 mutants (Adomas et al., 2014; Takaku et al., 2018), we performed *de novo* motif analysis to look at the enriched transcription factor binding motifs (Figure 4C). Interestingly, all mutant ChIP-seq peaks exhibited significantly enriched Forkhead motifs. ERE motifs were also enriched in Splice del (ranked 10th), C321fs (ranked second), and A333fs (ranked 13th, *p*-value = 1e-159) mutant ChIP-seq data. Since FOXA1 and ER-α are well-known as GATA3 co-factors (Kong et al., 2011; Theodorou et al., 2013), the enrichment of these co-factors’ motifs may indicate the importance of FOXA1 and ER-α for the chromatin binding activities of the GATA3 mutants (Adomas et al., 2014; Takaku et al., 2020). In addition to these co-factors, other transcription factor binding motifs (e.g., GRHL2, BORIS, TEAD4 AP2 gamma) were also enriched in the multiple data set. Importantly, the enriched GATA3 motifs identified by HOMER seem to lack the consensus motif DNA sequences (WGATAR) but show a partial motif, WGAT. To further confirm the partial motif enrichment, we performed the motif analysis by MEME (Machanick and Bailey, 2011). The MEME *de novo* motif analysis also showed a similar sequence enrichment, AGAT, at all three mutant peaks (Figure 4D). The center of each mutant peak aligned well with distribution of this non-canonical motif.

### GATA3 Truncation Mutants Alter GATA3 Chromatin Distribution

**FIGURE 5:**
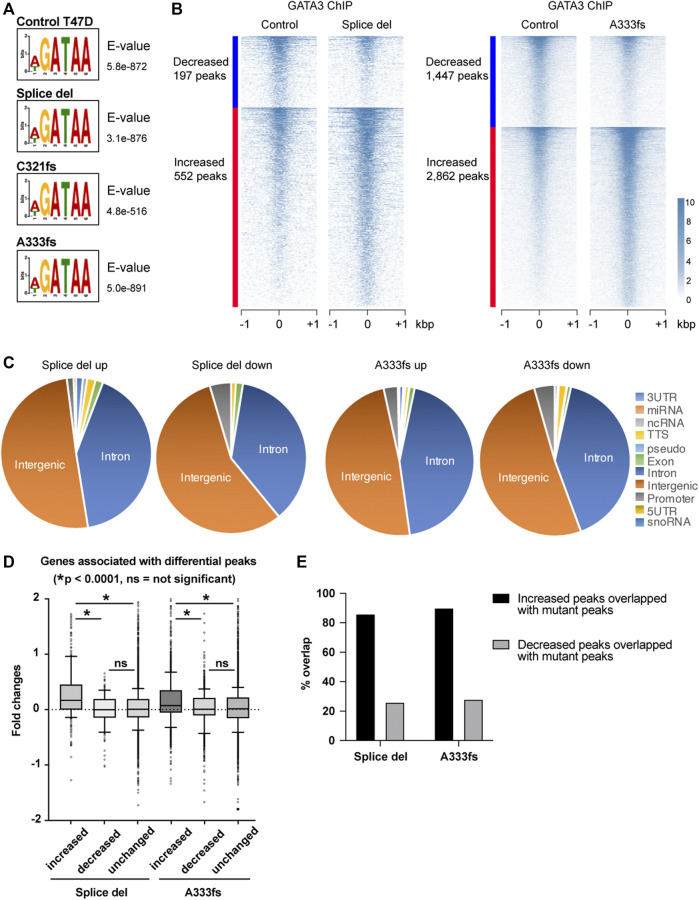
GATA3 re-distribution in GATA3 mutant cells. **(A)** The most significantly enriched motif by MEME ChIP analysis. **(B)** Heatmap showing decreased or increased GATA3 binding in Splice del (left) or A333fs mutant cells. **(C)** Genomic localization of differential peaks. **(D)** Correlation analysis of GATA3 differential peaks and gene expression. Increased, decreased, and unchanged peaks are assigned to nearest genes. Box plots show the fold changes of each GATA3 peak group associated genes. Fold changes were calculated between Splice del and control T47D cells or A333fs and control T47D cells. **(E)** Overlap between differential peaks and Splice del or A333fs peaks.

The established GATA3 mutant cell lines still express wild-type GATA3, which mimics the situation in breast tumors since most of the GATA3 mutations found in patients are heterozygous. To determine the impact of GATA3 mutant chromatin binding on the overall GATA3 distribution in T47D cells, we performed GATA3 ChIP-seq using the antibody that recognizes both wild-type and mutants (Figure 4A, Supplementary Figure S1B). In control T47D cells (GATA3 wild-type), the consensus GATA3 motif, WGATAA, was the most significantly enriched in MEME-ChIP analysis. Similarly, the most significant enriched sequence in the mutant cells was the same consensus motif, suggesting that the overall sequence preference of GATA3 is maintained in the presence of GATA3 truncation mutants. As shown in Figure 4A, there are frequent overlaps between Ty1 GATA3 mutant ChIP-seq and the total GATA3 ChIP-seq. We also observed Ty1 ChIP-seq unique peaks or total GATA3 ChIP-seq unique peaks (such as around the CDH1 transcription start site). These differential signals might be due to the relatively lower expression of the mutants compared to the wild-type GATA3 protein (Supplementary Figure S1B) or differences in antibody specificity. To identify differential GATA3 binding in the mutant cells, we first defined GATA3 peaks in each cell line by HOMER and performed the metaplot analysis at the GATA3 peaks. For the metaplot analysis, we generated the union GATA3 peak set (41,538 peaks) by merging all GATA3 peaks detected in control, Splice del, C321fs, and A333fs T47D cells. Metaplot analysis at the union GATA3 peaks indicates that weaker GATA3 enrichment in C321fs T47D cells and stronger GATA3 enrichment in Splice site or A333fs T47D cells compared to control T47D cells (Supplementary Figure S3A). To further identify the impacts of these mutants’ expression on the GATA3 binding, we performed EdgeR analysis. The total GATA3 binding was largely unchanged in Splice del, C321fs, and A333fs mutant expressing cells compared to the parental control T47D cells. At FDR <0.05 and absolute fold change >1.5, a small set of GATA3 peaks were defined as differential peaks in the mutant cells (Figure 5B, Supplementary Figure S3B–E). Among the three mutant cell lines, the A333fs mutant cell line had the most GATA3 differential binding events (∼10%) despite having the lowest expression level of the mutant (Figure 1B). Based on the EdgeR analysis with two biological replicates, 1,447 peaks showed decreased GATA3 ChIP-seq signals, while 2,862 peaks showed increased GATA3 ChIP-seq intensities in A333fs T47D cells (Figure 5B). On the other hand, with the same experimental setting, Splice del mutant expressed cells detected 197 decreased and 552 increased GATA3 binding sites compared to the control wild-type T47D cells. Many differential peaks were located at intergenic regions or introns (Figure 5C). To explore the association between differential peaks and gene expression, we assigned the differential peaks to the nearest genes to investigate the changes in gene expression between the mutant cells and control cells more closely. In both A333fs and Splice del mutant cells, increased GATA3 peaks were significantly associated with increased gene expression compared to decreased peaks or unchanged peaks, while decreased peaks didn’t exhibit a clear correlation with gene down-regulation (Figure 5D). To dissect the mechanism of differential GATA3 binding, we investigated the overlap between differential GATA3 peaks and the mutant-specific peaks defined by Ty1 ChIP-seq (Figure 5E). Most of the increased GATA3 peaks overlapped with Splice del mutant peaks in Splice del expressed cells and A333fs mutant peaks in A333fs mutant expressed cells. These data suggest that GATA3 truncation mutants influence GATA3 distributions in luminal breast cancer cells leading to differential gene expression.

## Discussion

Worldwide breast cancer genome profiling keeps revealing GATA3 as one of the primary targets for somatic mutations in breast cancer. The systemic analysis defined those GATA3 mutations as cancer drivers. However, our knowledge of GATA3 mutations in tumorigenesis, tumor progression, and acquisition of drug resistance is limited (Kouros-Mehr et al., 2006; Chou et al., 2010; Chou et al., 2013; Liu et al., 2016; Bi et al., 2020). We, and other groups, have previously reported that truncation mutations found around the ZnFn_2_ domain possess active roles in breast cancer properties and potentially stimulate tumor growth (Usary et al., 2004; Mair et al., 2016; Gustin et al., 2017; Emmanuel et al., 2018; Takaku et al., 2018; Takaku et al., 2020). The distribution of GATA3 patient mutations is not focal but widely spread in the C-terminal region of the GATA3 gene (Ciriello et al., 2015; Pereira et al., 2016). Some of them, including splice site mutations, completely lack the wild-type sequence of the ZnFn_2_ domain (Takaku et al., 2015). While other frameshift mutations partially or fully have the ZnFn_2_ domain (Figure 1A). Therefore, it is still unclear whether these slight differences in the GATA3 protein structure can produce different outcomes in luminal breast cancer.

In this study, using our newly established stable cell lines, we analyzed the function of three truncation mutants, X308 splice site deletion (Splice del), C321fs, and A333fs in T47D cells. Transcriptome analysis revealed that although each mutant has a distinct impact on a subset of genes, many differentially expressed genes (>50%) are commonly up- or down-regulated in at least two of the three GATA3 mutant cell lines. Among them, EMT-related genes showed similar alterations across three GATA3 mutant cell lines. We previously examined the impacts of a similar truncation mutant R330fs in the same T47D cells using an exogenous expression model as well as an endogenous expression system by CRISPR Cas9 genome editing (Takaku et al., 2018). Similar to Splice del, C321fs, and A333fs mutations, R330fs mutant expression induced altered expression of the EMT genes such as TWIST1. The function of another truncation mutant, D336fs, was also reported by our group and other groups (Adomas et al., 2014; Gustin et al., 2017; Emmanuel et al., 2018). Both R330fs and D336fs expression stimulated tumor growth of estrogen receptor-positive breast cancer cells in the mouse xenograft model. Therefore, GATA3 splice site or frameshift mutations that partially or fully alter amino acid residues of the ZnFn_2_ domain may have similar impacts on breast cancer properties and interrupt luminal transcriptome.

To further identify how GATA3 truncation mutants modulate gene expression, we performed GATA3 mutant specific ChIP-seq and total GATA3 (wild-type and mutant) ChIP-seq. Many mutant binding sites were overlapped across Splice del, C321fs, and A333fs ChIP-seq data. We also observed differential binding sites, particularly between Splice del and A333fs mutants. C321fs ChIP-seq data showed fewer peaks compared to the other mutants. These results may suggest slightly different chromatin binding affinities, protein stability, and/or DNA sequence preference. The motif analysis at the mutant peaks revealed a unique motif enrichment, AGAT, which differs from the consensus motif, WGATAA enriched at the total GATA3 ChIP-seq peaks. Interestingly, the R330fs mutant ChIP-seq data also showed similar non-canonical motif enrichment. These results suggest that the partial or complete disruption of the second zinc-finger domain results in the non-conventional chromatin binding of the GATA3 mutants. Based on the total GATA3 ChIP-seq data, such an altered binding specificity contributes to differential GATA3 binding in the GATA3 mutant cells. The motif analysis of the mutants also showed significant enrichment of the GATA3 well-known co-factors, FOXA1 and Estrogen Receptor alpha (ER-α). Both R330fs and X308 splice site mutants were previously reported to influence the distributions of these GATA3 co-factors. Thus, the enrichment of the co-factors’ motifs suggests that Splice del, C321fs and A333fs may alter FOXA1 and/or ER-α localization (Hruschka et al., 2020).

In addition to observing multiple common events between different types of GATA3 mutations, distinct phenotypes were also observed. For instance, in RNA-seq data, C321fs and A333fs cells exhibited enrichment in cell junction-related pathways, while Splice del mutant cells did not show any enrichment. 10,859 A333fs peaks were not overlapped with Splice del mutant peaks. In the case of R330fs, there was a significant reduction of progesterone receptor (PR) expression, but such reduction of PR expression was not detected in Splice del, C321fs, nor A333fs mutant T47D cells. Further investigation is essential for understanding the common and unique roles of GATA3 mutations in breast cancer. It remains elusive if GATA3 can be a therapeutic target or if these GATA3 mutant breast cancer cells are sensitive to specific chemicals (Mair et al., 2016).

## Data Availability

The datasets presented in this study can be found in online repositories. The names of the repository/repositories and accession number(s) can be found below: GEO: GSE190381.
