# Supplementary figures and images for: GATA3 Truncation Mutants Alter EMT Related Gene Expression via Partial Motif Recognition in Luminal Breast Cancer Cells
Source: Front Genet. 2022 Jan 28;13:820532. doi: 10.3389/fgene.2022.820532 (PMC8831884; doi:10.3389/fgene.2022.820532)

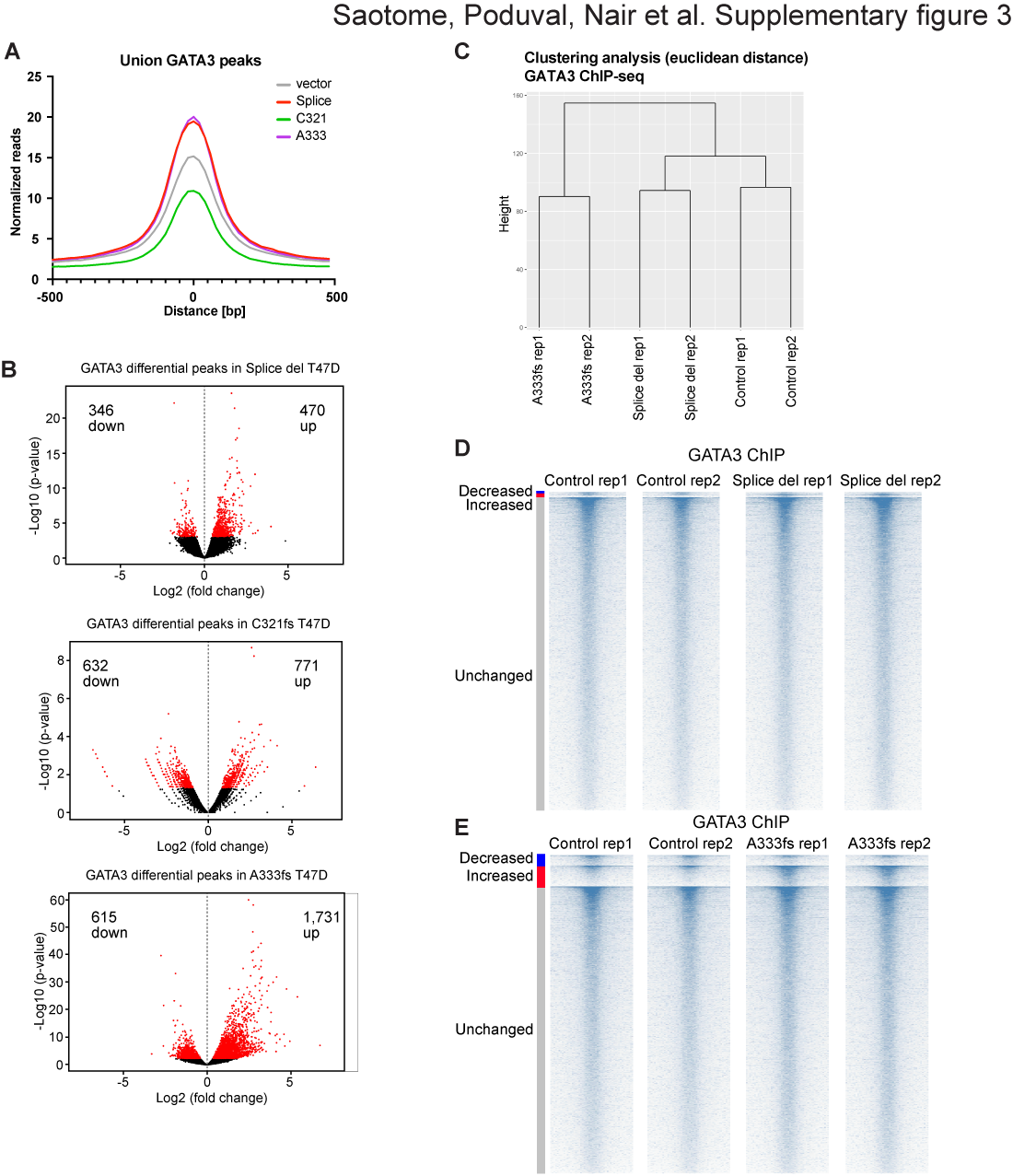

Supplement: Supplementary file 1 [file Image3.tif]

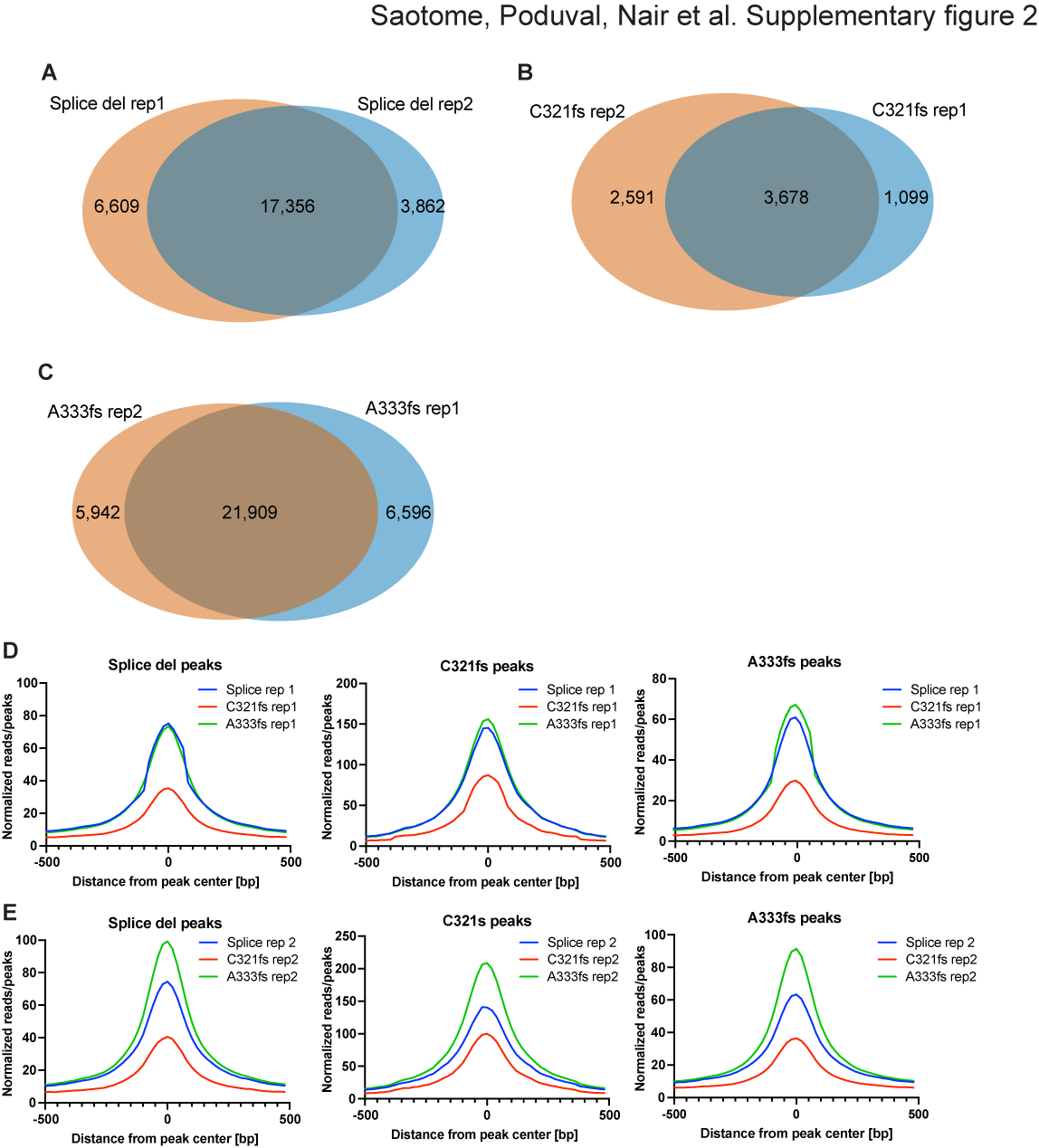

Supplement: Supplementary file 2 [file Image2.tif]

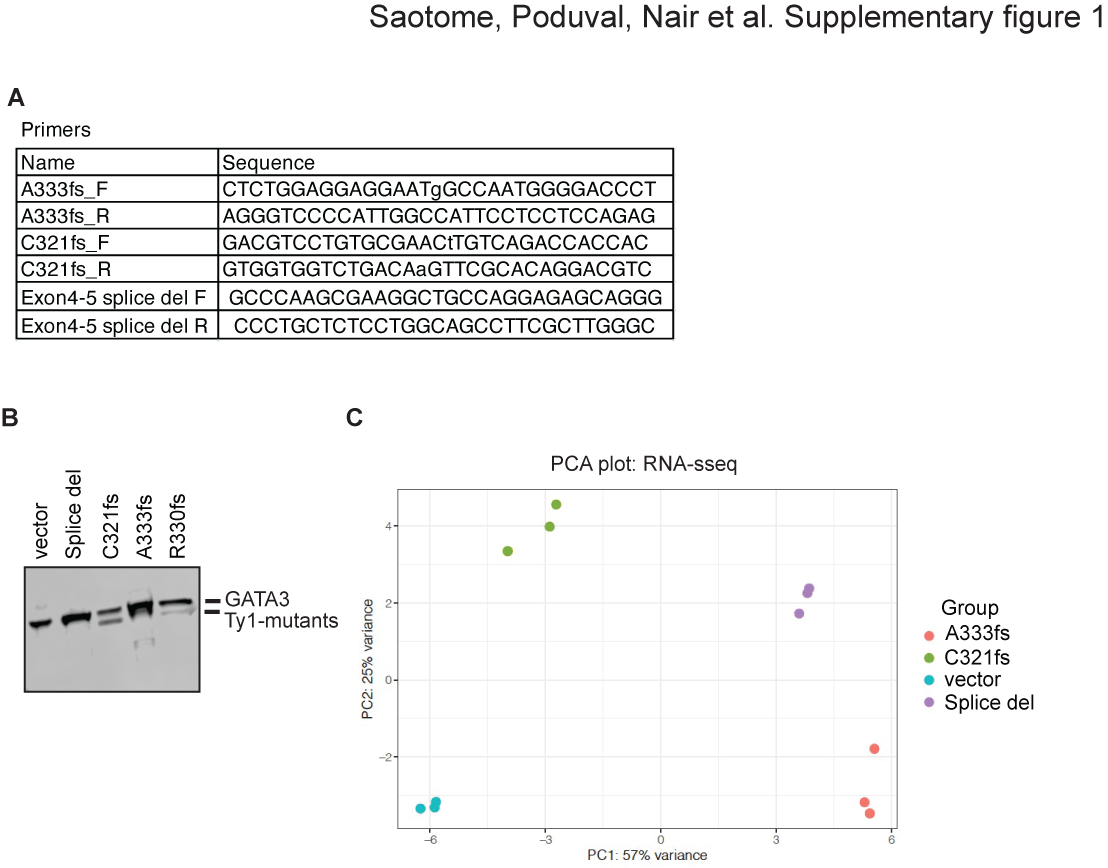

Supplement: Supplementary file 3 [file Image1.tif]
