# Supplementary material for: GATA3 Truncation Mutants Alter EMT Related Gene Expression via Partial Motif Recognition in Luminal Breast Cancer Cells
Source: Front Genet. 2022 Jan 28;13:820532. doi: 10.3389/fgene.2022.820532 (PMC8831884; doi:10.3389/fgene.2022.820532)
Supplement: Supplementary file 4 [file DataSheet2.docx]

**Supplementary Figure 1. Generation of GATA3 mutant T47D cells**

**(A)** Primers used in the study. The indicated primers were used to generate Splice site del, C321fs, and A333fs mutant expression vectors. **(B)** Western blot showing GATA3 wild-type and mutants. Anti-GATA3 antibody (Cell Signaling, D13C9) was used to detect wild-type and mutant GATA3. **(C)** PCA plot showing the similarity of RNA-seq data in control (empty) T47D cells and GATA3 mutant cells.

**Supplementary Figure 2. Data reproducibility of GATA3 mutant ChIP-seq**

**(A)** Venn diagram showing Splice del mutant peaks from two biological replicates. The same uniquely-mapped reads (15 million reads) were used to define peaks by HOMER. **(B)** Venn diagram showing C321fs mutant peaks from two biological replicates. **(C)** Venn diagram showing A333fs mutant peaks from two biological replicates. **(D-E)** Metaplot showing Ty1 ChIP-seq signals at GATA3 mutant peaks. Splice del (left), C321fs (middle), or A333fs (right) peaks were used to measure normalized reads per peak in each cell line. The common (overlapped) peaks between biological replicates were used for metaplot analyses. The data from replicates 1 and 2 are shown in (D) and (E) respectively.

**Supplementary Figure 3. GATA3 peak analysis in GATA3 mutant lines**

**(A)** Metaplot showing GATA3 ChIP-seq signals in each T47D cell line at the union GATA3 peaks. GATA3 peaks in wild-type (vector) T47D cells or GATA3 mutant expressing T47D cells were identified by HOMER. To obtain GATA3 union peaks, the overlapped GATA3 peaks were merged, and unique peaks (non-overlapped peaks) were retained. **(B)** Volcano plot showing differential peaks. Pairwise comparison using individual GATA3 ChIP-seq data in each cell line was performed. Differential peaks were defined by EdgeR with FDR < 0.05 and absolute fold change > 1.5. **(C)** Clustering analysis of GATA3 ChIP-seq data. **(D)** Heatmap showing up-, down-, and unchanged GATA3 peaks in Splice del cells. GATA3 ChIP-seq data from two biological replicates were used to define differential peaks. **(E)** Heatmap showing up, down, and unchanged GATA3 peaks in A333fs cells. Differential peaks were identified by EdgeR with two replicates.
